# Comparative analysis on survival and tissue damage of different environmental stress factors in Pacific white shrimp *Litopenaeus vannamei*

**DOI:** 10.1016/j.cirep.2025.200219

**Published:** 2025-03-13

**Authors:** Lulu Han, Peiyu Yan, Mengqiang Wang

**Affiliations:** a MOE Key Laboratory of Marine Genetics and Breeding, Shandong Key Laboratory of Marine Seed Industry (preparatory), and Qingdao Institute of Maritime Silk Road (Qingdao Institute of Blue Seed Industry), Ocean University of China, Qingdao 266003, PR China; b Hainan Key Laboratory of Tropical Aquatic Germplasm, Sanya Oceanographic Institution, Ocean University of China, Sanya 572024, PR China; c Southern Marine Science and Engineering Guangdong Laboratory (Guangzhou), Guangzhou 511458, PR China

**Keywords:** *Litopenaeus vannamei*, Ammonia stress, Nitrite stress, Sulfide stress, Histological analysis

## Abstract

Ammonia, nitrite and sulfide are aquatic systemic toxicants that threaten the health of aquatic animals. They directly affect aquatic animal survival and aquaculture productivity, and cause damage to aquatic animal tissues. This experiment investigated the effects of differences in concentrations and types of toxicants in aquatic systems on the survival rate of shrimp and the degree of damage to various tissues, which will provide a reference to the maintenance of the water quality conditions in shrimp aquaculture process. All the three chemicals affected shrimp survival compared to the control, and there was no significant difference between the effects of different concentrations of ammonia (10 mg/L, 20 mg/L and 30 mg/L ammonia-N) on survival at the end of stress, while both nitrite (20 mg/L, 40 mg/L and 60 mg/L nitrite-N) and sulfide (2 mg/L, 3 mg/L and 4 mg/L sulfide) gradually decreased the survival rate at the end of stress with increasing stress concentration. Ammonia and sulfide gradually increased the damage to the hepatopancreas with increasing stress concentrations, and all the three concentrations of nitrite caused severe damage to the hepatopancreas. The separation of the epithelial cells of the midgut from the basement membrane was more pronounced with increasing stress concentrations of all the three chemicals. Dissociation of muscle tissue was more severe at higher concentrations of ammonia and sulfide, and severe dissociation of muscle occurred at all the three concentrations of nitrite. All the three chemicals showed progressively more damage to gill with increasing stress concentrations. In summary, survival and tissue damage to *Litopenaeus vannamei* progressively increased with increasing concentrations of ammonia, nitrite, and sulfide, with nitrite overall being more damaging to survival and tissue than ammonia and sulfide.

## Highlights

• Comparison of the effects of different environmental stress factors on the survival and tissue damage of *Litopenaeus vannamei.*
• Damage to the hepatopancreas, midgut, muscles and gills progressively increased with increasing concentrations of ammonia, nitrite and sulfide.
• Overall, nitrite (20 mg/L, 40 mg/L, 60 mg/L) caused more damage than ammonia (10 mg/L, 20 mg/L, 30 mg/L) and sulfide (2 mg/L, 3 mg/L, 4 mg/L).

## Introduction

The Pacific white shrimp (*Litopenaeus vannamei*) plays an extremely important role in aquaculture [1]. In recent years, shrimp aquaculture has experienced economic losses caused by diseases [2], which are caused by complex interactions between the host, the environment and pathogens, and environmental stresses often act as a predisposing factor. Aquatic systems contain a wide range of toxic substances such as heavy metals [3,4], ammonia, nitrite, and sulfide. These toxic substances accumulate in aquatic organisms [5], causing immune and physiological responses [6] and increasing susceptibility to pathogens [7].

Ammonia nitrogen is a key indicator for water management, and in aquatic environments it may originate from the decomposition of food residues and excrement [8]. Excessive ammonia nitrogen in the water inhibits growth performance and increases molting frequency in aquatic animals [9], as well as damaging the gills and hepatopancreas of shrimps [10], affecting the antioxidant system and respiratory metabolism [[11], [12], [13]]. Accumulation of residual bait and feces during shrimp culture and poor water circulation can lead to elevated nitrite concentrations in culture water, even as high as 20 mg/L [14]. Nitrite stress can lead to a number of problems such as abnormal growth and increased mortality in shrimp [15,16]. Nitrite stress also causes hepatopancreatic cell shrinkage, lysis and vacuolization and induces oxidative damage to the hepatopancreas in shrimp, which can increase the risk of apoptosis [17,18]. Sulfides are produced under anaerobic conditions through the decomposition of organic matter and the reduction of sulfates, and are typically found in the substrate and sediments of aquatic environments. Lower sulfide tolerance in crustaceans compared to other benthic invertebrates [19]. Sulfide stress disrupts the structural integrity of gill and gut tissues and triggers an immune response, as well as affecting osmoregulation and antimicrobial capacity [20].

In this experiment, by comparing the survival and the degree of tissue damage of L. *vannamei* under different concentrations of different toxicants, we obtained the differences between the concentrations and types of toxicants (ammonia, nitrite and sulfide) in aquatic systems on the survival rate of shrimp and the damage of various tissues, which will provide a reference for the maintenance of the water quality conditions in the shrimp aquaculture process.

## Materials and methods

### Shrimp and culture conditions

L. *vannamei* were obtained from the farm of Hainan Zhongzheng Aquatic Technology Co., Ltd. The shrimp had normal stamina and vitality and were sampled and tested without disease. The weight of the shrimp was 12.77 ± 2.76 g, the salinity of the seawater was 3 %, the pH was about 8.2, the temperature was 24.7 ± 0.29 °C.

### Stress exposure and shrimp sampling

After transient rearing, based on previous experimental studies on the stress concentrations of three different chemicals, including ammonia, nitrite, and sulfide, in L. *vannamei* [[21], [22], [23]], the shrimps were divided into ten groups of 80 shrimp each, which were control group, A_1_ group (10 mg/L ammonia-N), A_2_ group (20 mg/L ammonia-N), A_3_ group (30 mg/L ammonia-N), Y_1_ group (20 mg/L nitrite-N), Y_2_ group (40 mg/L nitrite-N), Y_3_ group (60 mg/L nitrite-N), S_1_ group (2 mg/L sulfide), S_2_ group (3 mg/L sulfide), S_3_ group (4 mg/L sulfide). The stock solution of 10 g/L ammonia-N was prepared by dissolving 38.21 g of NH_4_Cl in 1 L of distilled water. The stock solution of 10 g/L nitrite-N was prepared by dissolving 49.29 g of NaNO_2_ in 1 L of distilled water. The stock solution of 1 g/L sulfide was prepared by dissolving 7.49 g Na_2_S·9H_2_O in 1 L of distilled water. Half of the seawater was renewed with the same concentration of seawater every 12 h, and the concentrations of ammonia nitrogen, nitrite nitrogen and sulfide in seawater were detected in time using spectrophotometry. The cumulative number of surviving shrimps in each stress group was counted every 12 h and dead shrimp were removed in time. No food was provided during 132 h of ammonia, nitrite, and sulfide stress. At the end of 132 h of stress, the hepatopancreas, midgut, muscle and gills of four shrimps from each group were sampled for histological analysis.

### Histological analysis

The hepatopancreas, midgut, muscle, and gills were harvested at the end of the stress period. Subsequently, these tissues were subjected to a series of procedures such as fixation, dehydration, transparency and wax impregnation, and finally embedded in paraffin wax. Samples were sliced to a thickness of 5 μm with a microtome (HistoCore Nanocut R, Leica, Germany) and paraffin was removed in a xylene bath and rehydrated in successive baths of 95 % to 30 % ethanol. Tissue sections were stained with hematoxylin and eosin (HE), fixed with neutral balsam and the sections were observed under a light microscope and photographed (DM2500 LED, Leica, Germany).

### Statistical analysis

Graphpad Prism 9 software was used to graph and analyze the relevant data. Each stress factor consisted of three treatment groups with different concentrations and one control group, and the total survival data of 80 shrimps in each group were analyzed using the Log-Rank method. Data were considered significantly different from each other if P≤ 0.05.

## Results

### Cumulative survival of L. *vannamei* at different concentrations of stress factors

**Fig. 1:**
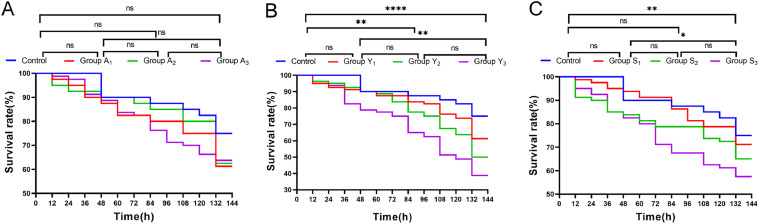
Cumulative survival curves of L. *vannamei* under different environmental stress factors.

The cumulative survival of L. *vannamei* under different concentrations of ammonia nitrogen stress is shown in Fig. 1A. The cumulative survival rates of L. *vannamei* exposed to all the three ammonia concentrations (A_1_ group: 10 mg/L ammonia-N, A_2_ group: 20 mg/L ammonia-N, A_3_ group: 30 mg/L ammonia-N) gradually declined as the stress time increased. Notably, a significant reduction in survival was observed between 120 h and 132 h in groups A_1_ and A_2_. The survival rates at the end of stress were 75 % in the control group, 61.25 % in the A_1_ group, 62.5 % in the A_2_ group, and 63.75 % in the A_3_ group, and there was no significant difference in the cumulative survival rate at the end of stress among the three treatment groups with different concentrations and it was significantly lower than that of the control group.

The cumulative survival of L. *vannamei* under different concentrations of nitrite stress is shown in Fig. 1B. The cumulative survival rates of L. *vannamei* exposed to all the three nitrite concentrations (Y_1_ group: 20 mg/L nitrite-N, Y_2_ group: 40 mg/L nitrite-N, Y_3_ group: 60 mg/L nitrite-N) gradually declined as the stress time increased. Notably, a significant reduction in survival was observed in all the three groups between 120 and 132 hours. As the nitrite concentration increased, the greater the difference between the stress groups and the control group, with both the Y_2_ and Y_3_ groups being significantly different from the control group. Survival rates at the end of stress were 75 % in the control group, 61.25 % in the Y_1_ group, 50.00 % in the Y_2_ group, and 38.75 % in the Y_3_ group, showing a trend of decreasing cumulative survival with increasing nitrite concentration.

The cumulative survival of L. *vannamei* under different concentrations of sulfide is shown in Fig. 1C. As the stress time increased, the cumulative survival rates of L. *vannamei* exposed to all the three sulfide concentrations (S_1_ group: 2 mg/L nitrite-N, S_2_ group: 3 mg/L nitrite-N, S_3_ group: 4 mg/L nitrite-N) exhibited a declining trend. Notably, the survival rates in the S_1_ and S_2_ groups decreased significantly between 120 and 132 hours. The S_3_ group was significantly different from both the control and S_1_ groups. Survival rates at the end of stress were 75 % in the control group, 71.25 % in the S_1_ group, 65.00 % in the S_2_ group, and 57.50 % in the S_3_ group, and the cumulative survival rate decreased progressively with increasing sulfide concentration.

### Morphology of hepatopancreatic tissues after stress with various chemicals

**Fig. 2:**
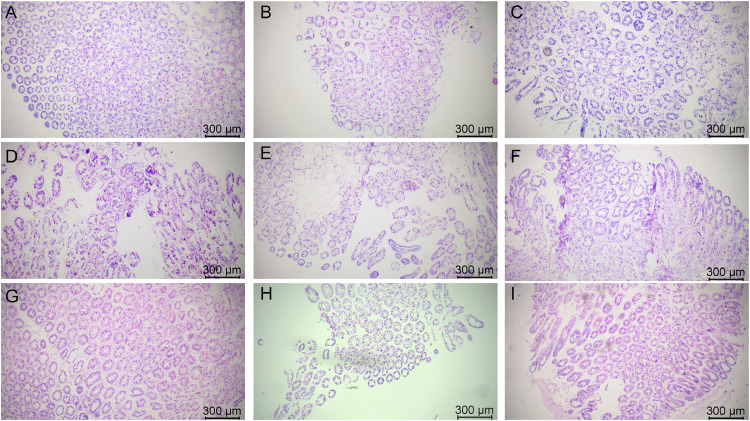
Hepatopancreatic tissue changes in L. *vannamei* after 132 h of exposure (50× magnification). (A) Group A_1_ (10 mg/L ammonia-N); (B) Group A_2_ (20 mg/L ammonia-N); (C) Group A_3_ (30 mg/L ammonia-N); (D) Group Y_1_ (20 mg/L nitrite-N); (E) Group Y_2_ (40 mg/L nitrite-N); (F) Group Y_3_ (60 mg/L nitrite-N); (G) Group S_1_ (2 mg/L sulfide); (H) Group S_2_ (3mg/L sulfide); (I) Group S_3_ (4mg/L sulfide).

**Fig. 3:**
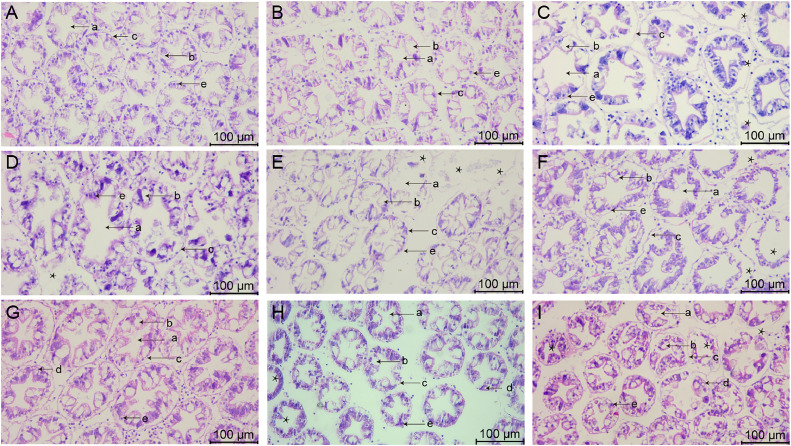
Hepatopancreatic tissue changes in L. *vannamei* after 132 h of exposure (200× magnification). (A) Group A_1_ (10 mg/L ammonia-N); (B) Group A_2_ (20 mg/L ammonia-N); (C) Group A_3_ (30 mg/L ammonia-N); (D) Group Y_1_ (20 mg/L nitrite-N); (E) Group Y_2_ (40 mg/L nitrite-N); (F) Group Y_3_ (60 mg/L nitrite-N); (G) Group S_1_ (2 mg/L sulfide); (H) Group S_2_ (3mg/L sulfide); (I) Group S_3_ (4mg/L sulfide). (a) Lumen of hepatopancreatic tubule; (b) B-cells secretory cell; (c) Basement membrane; (d) Restzellen cells; (e) E-cells embryonic cell. Asterisks indicate vacuolization and shrinkage.

The morphology of hepatopancreatic tissues stained by HE is shown in Fig. 2, Fig. 3. Hepatopancreas in group A_1_ were tightly arranged, with a few cells showing vacuolization (Fig. 2A), and R-cells were blurred and difficult to identify (Fig. 3A). In group A_2_, some hepatopancreatic cells were deformed and fragmented (Fig. 2B), and both the hepatic tubules and the gap between the cells appeared to be enlarged (Fig. 3B). In group A_3_, the hepatopancreatic cells were severely atrophied and deformed, and most of the cells underwent lysis, and the complete cellular morphology could not be observed (Fig. 2C). The structure of the hepatic tubules was severely disrupted, while the basement membrane appeared distinctly separated from the epithelium, and many hematopoietic cells infiltrated the damaged tissues (Fig. 3C).

Hepatopancreatic cells in group Y_1_ showed extensive ulceration (Fig. 2D), and it was difficult to clearly observe the structure of the epithelial cells, and no clear boundaries between the cells could be observed (Fig. 3D). In group Y_2_, connective tissue between hepatopancreatic vacuoles was damaged, and several vacuoles were fused into one large vacuole (Fig. 2E), and the epithelium could not be observed in some of the cells, only the edge of the basement membrane was observed (Fig. 3E). Hepatopancreatic cells in group Y_3_ showed extensive necrosis and most of the cells appeared deformed and blurred (Fig. 2F). The structure of hepatic tubules was severely damaged and enlarged significantly, and the number of B-cells were significantly reduced (Fig. 3F).

In group S_1_, most of the hepatopancreas cells were well aligned, and a few cells showed atrophy and deformation (Fig. 2G). Some of the hepatic tubules appeared slightly enlarged and a few blood cells were present in the tissue interstitium (Fig. 3G). Hepatopancreatic cells in group S_2_ were loosely arranged and most of the cells showed vacuolization (Fig. 2H), while R-cells were blurred and a small number of hemocytes distributed in the intercellular spaces (Fig. 3H). Hepatopancreatic cells in group S_3_ were severely damaged, with extensive necrosis and deformation (Fig. 2I), severe cytolysis and fragmentation, and fragmented cell tissues could be observed in the interstitial space and lumen of the hepatic tubules (Fig. 3I).

### Morphology of midgut tissues after stress with various chemicals

**Fig. 4:**
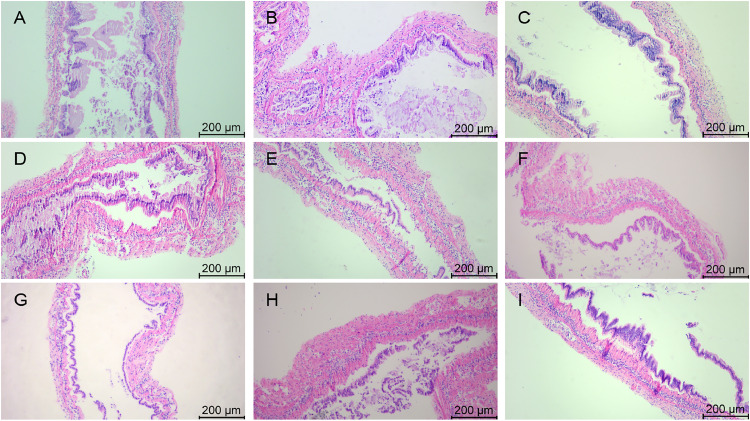
Midgut tissue changes in L. *vannamei* after 132 h of exposure (100× magnification). (A) Group A_1_ (10 mg/L ammonia-N); (B) Group A_2_ (20 mg/L ammonia-N); (C) Group A_3_ (30 mg/L ammonia-N); (D) Group Y_1_ (20 mg/L nitrite-N); (E) Group Y_2_ (40 mg/L nitrite-N); (F) Group Y_3_ (60 mg/L nitrite-N); (G) Group S_1_ (2 mg/L sulfide); (H) Group S_2_ (3mg/L sulfide); (I) Group S_3_ (4mg/L sulfide).

**Fig. 5:**
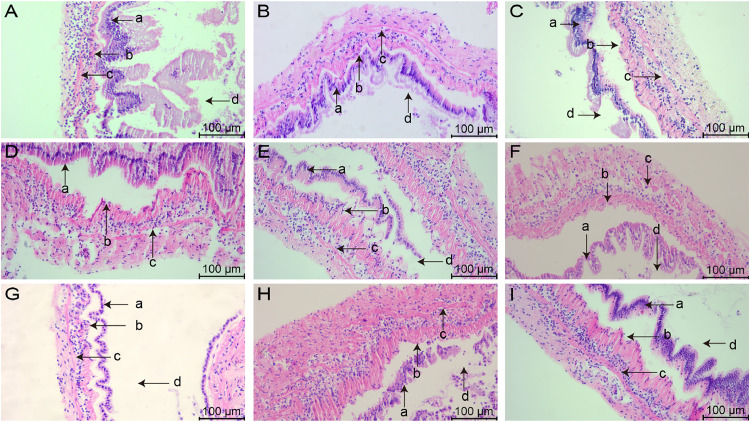
Midgut tissue changes in L. *vannamei* after 132 h of exposure (200× magnification). (A) Group A_1_ (10 mg/L ammonia-N); (B) Group A_2_ (20 mg/L ammonia-N); (C) Group A_3_ (30 mg/L ammonia-N); (D) Group Y_1_ (20 mg/L nitrite-N); (E) Group Y_2_ (40 mg/L nitrite-N); (F) Group Y_3_ (60 mg/L nitrite-N); (G) Group S_1_ (2 mg/L sulfide); (H) Group S_2_ (3mg/L sulfide); (I) Group S_3_ (4mg/L sulfide). (a) Intestinal epithelial cells; (b) Basement membrane; (c) Circular muscle; (d) Lumen.

The morphology of midgut tissues stained by HE is shown in Fig. 4, Fig. 5. In group A_1_, the epithelial cell junctions were broken and fragmented (Fig. 4A), along with a slight detachment from the basement membrane and the presence of nuclei in the intestinal lumen (Fig. 5A). In group A_2_, there was localized necrosis of the epithelial cells, accompanied by shedding of some of the cells (Fig. 4B), and the nuclei appeared to be solidified (Fig. 5B). The epithelial cells in group A_3_ were sparsely arranged (Fig. 4C) and clearly separated from the basement membrane (Fig. 5C).

In group Y_1_, some of the epithelial cells were necrotic and fractured (Fig. 4D), and the basement membrane appeared to have a gap with the epithelial cells (Fig. 5D). In group Y_2_, the epithelial cells were deformed in folded form and appeared in the intestinal lumen (Fig. 4E), and the nuclei appeared agglutinated (Fig. 5E). The epithelial cells in group Y_3_ underwent detachment and curling (Fig. 4F), and the cells showed vacuolization and obvious separation (Fig. 5F).

The connection between the epithelial cells in the midgut of group S_1_ was impaired (Fig. 4G), the cell layer became thin and loosely connected, as well as detached from its original position (Fig. 5G). Some epithelial cells in the S_2_ group were necrotic and the vacuoles were ruptured (Fig. 4H), and some cells were dispersed in the intestinal lumen (Fig. 5H). Epithelial cells in group S_3_ underwent junctional breaks (Fig. 4I), contraction and deformation, and increased and aggregated nuclei (Fig. 5I).

### Morphology of muscle tissues after stress with various chemicals

**Fig. 6:**
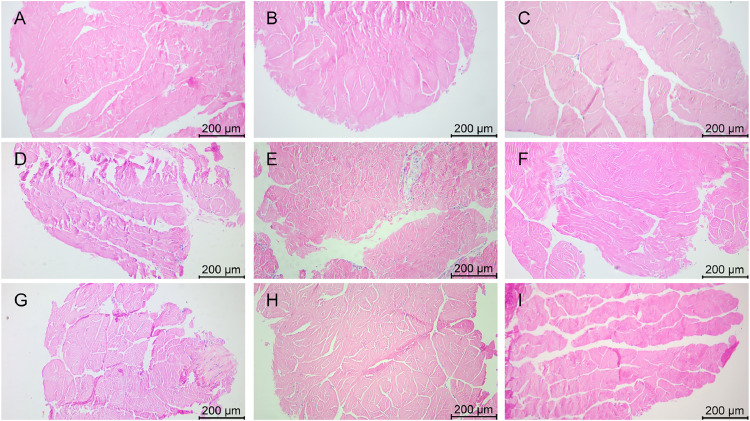
Muscle tissue changes in L. *vannamei* after 132 h of exposure (100× magnification). (A) Group A_1_ (10 mg/L ammonia-N); (B) Group A_2_ (20 mg/L ammonia-N); (C) Group A_3_ (30 mg/L ammonia-N); (D) Group Y_1_ (20 mg/L nitrite-N); (E) Group Y_2_ (40 mg/L nitrite-N); (F) Group Y_3_ (60 mg/L nitrite-N); (G) Group S_1_ (2 mg/L sulfide); (H) Group S_2_ (3mg/L sulfide); (I) Group S_3_ (4mg/L sulfide).

**Fig. 7:**
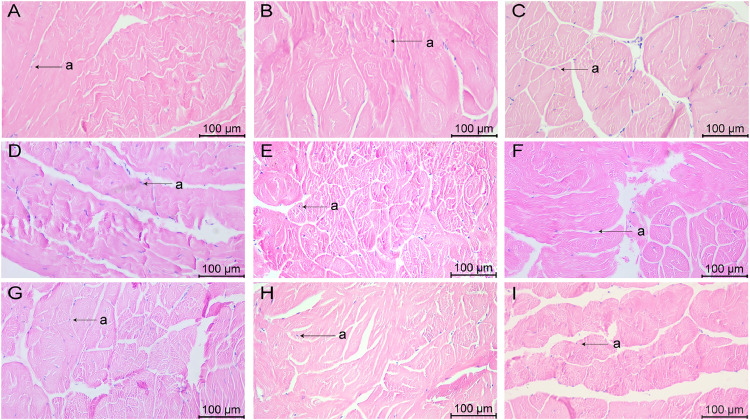
Muscle tissue changes in L. *vannamei* after 132 h of exposure (200× magnification). (A) Group A_1_ (10 mg/L ammonia-N); (B) Group A_2_ (20 mg/L ammonia-N); (C) Group A_3_ (30 mg/L ammonia-N); (D) Group Y_1_ (20 mg/L nitrite-N); (E) Group Y_2_ (40 mg/L nitrite-N); (F) Group Y_3_ (60 mg/L nitrite-N); (G) Group S_1_ (2 mg/L sulfide); (H) Group S_2_ (3mg/L sulfide); (I) Group S_3_ (4mg/L sulfide). (a) nuclear.

The morphology of the HE-stained muscle tissue is shown in Fig. 6, Fig. 7, with the pink area being the muscle fibers and the purple part being the nuclei. Separation of muscle tissue occurred in group A_1_, with muscle fiber breakage (Fig. 6A) and nuclear agglutination (Fig. 7A). The muscle fibers in group A_2_ were disorganized (Fig. 6B) and the nuclei were unevenly distributed (Fig. 7B). In group A_3_, tissue separation was obvious and the spacing between adjacent muscle bundles was large (Fig. 6C), and some cell nuclei appeared in the tissue gap (Fig. 7C).

In group Y_1_, the local muscle tissue was necrotic (Fig. 6D), while the transverse muscle was fractured and the nuclei appeared enlarged (Fig. 7D). A large separation between the tissues of group Y_2_ occurred (Fig. 6E), with muscle fibers breaking into clumps and transverse striations in the fibers being illegible (Fig. 7E). In group Y_3_, the muscle tissue was shattered and fractured (Fig. 6F). The muscle fibers were broken into fragments, with distinct gaps between adjacent muscle bundles and uneven distribution of nuclei (Fig. 7F).

In group S_1_, some muscle tissues were dissociated and myofibers were fragmented (Fig. 6G), and the morphology of some cell nuclei was deformed (Fig. 7G). In the S_2_ group, spacing appeared between adjacent muscle bundles (Fig. 6H), while cell nuclei appeared between damaged tissues (Fig. 7H). The tissues of group S_3_ were fractured and broken into pieces, and the tissue structure became disorganized (Fig. 6I). Most of the transverse lines in the fibers disappeared, and the distribution of cell nuclei was scattered (Fig. 7I).

### Morphology of gill tissues after stress with various chemicals

**Fig. 8:**
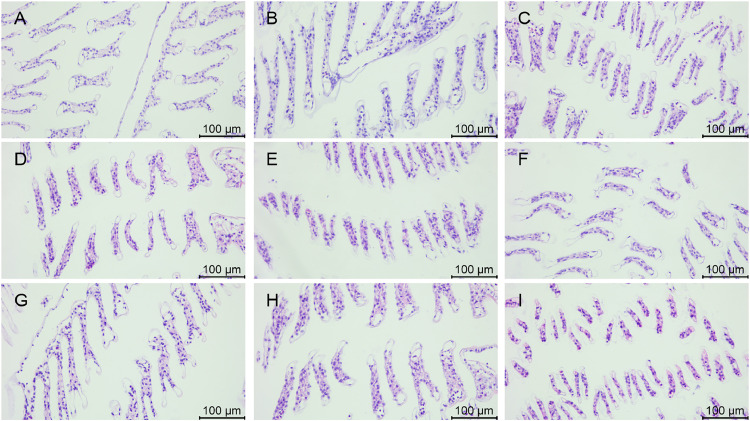
Gill tissue changes in L. *vannamei* after 132 h of exposure (200× magnification). (A) Group A_1_ (10 mg/L ammonia-N); (B) Group A_2_ (20 mg/L ammonia-N); (C) Group A_3_ (30 mg/L ammonia-N); (D) Group Y_1_ (20 mg/L nitrite-N); (E) Group Y_2_ (40 mg/L nitrite-N); (F) Group Y_3_ (60 mg/L nitrite-N); (G) Group S_1_ (2 mg/L sulfide); (H) Group S_2_ (3mg/L sulfide); (I) Group S_3_ (4mg/L sulfide).

**Fig. 9:**
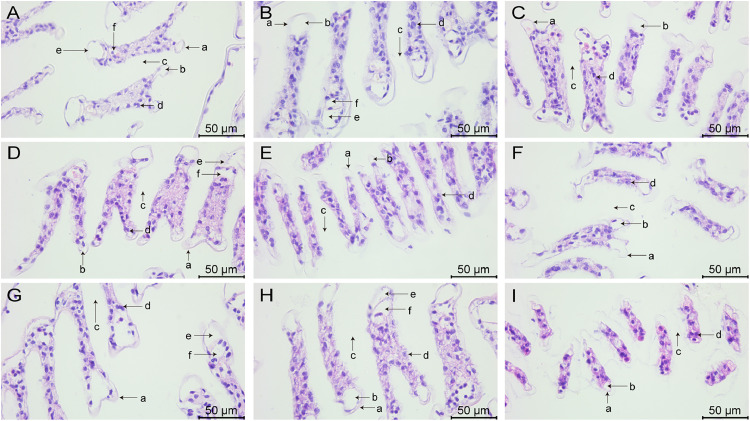
Gill tissue changes in L. *vannamei* after 132 h of exposure (400× magnification). (A) Group A_1_ (10 mg/L ammonia-N); (B) Group A_2_ (20 mg/L ammonia-N); (C) Group A_3_ (30 mg/L ammonia-N); (D) Group Y_1_ (20 mg/L nitrite-N); (E) Group Y_2_ (40 mg/L nitrite-N); (F) Group Y_3_ (60 mg/L nitrite-N); (G) Group S_1_ (2 mg/L sulfide); (H) Group S_2_ (3mg/L sulfide); (I) Group S_3_ (4mg/L sulfide). (a) Cuticle; (b) Epithelial cells; (c) Diaphragm; (d) Blood cells; (e) entering gill vessels; (f) exiting gill vessels.

The morphology of gill tissues stained by HE is shown in Fig. 8, Fig. 9. In group A_1_, the main gill shafts and neatly arranged gill filaments were observed and the gill filaments were slightly constricted (Fig. 8A), while the entering and exiting gill vessels were clearly visible (Fig. 9A). A few gill filaments in group A_2_ showed deformation (Fig. 8B) and increased number of hemocytes (Fig. 9B). In group A_3_, the arrangement of gill filaments was twisted and disorganized with obvious constriction (Fig. 8C), thinning of cuticle and blurring of gill vessels (Fig. 9C).

Some of the gill filaments in group Y_1_ underwent bending and contraction (Fig. 8D), and some of the gill filaments could be observed with entering and exiting gill vessels (Fig. 9D). In group Y_2_, gill filaments contracted significantly (Fig. 8E), cells appeared to be necrotic, and cuticle rupture occurred (Fig. 9E). The gill filaments of group Y_3_ were dispersed and severely deformed (Fig. 8F). The cuticle was thinned and the gill vessels were severely damaged with luminal vacuolation (Fig. 9F).

In group S_1_, the gill filaments were neatly arranged and tightly packed with a few gill filaments showing constriction and deformation (Fig. 8G), and the gill vessels were clearly visible (Fig. 9G). Some gill filaments in group S_2_ showed slight disorganization in their arrangement (Fig. 8H), and some gill filaments underwent contraction and the cuticle appeared to be ruptured (Fig. 9H). In group S_3_, the gill filaments were arranged in a twisted and disordered manner, with loose connections, obvious constriction (Fig. 8I), severe deformation of the gill vessels, and an increase in the number of hemocytes (Fig. 9I).

## Discussion

### Effects of various concentrations of different chemicals on the survival of L. *vannamei*

In the present study, the effects of different concentrations of ammonia, nitrite and sulfide on the survival of shrimp were investigated based on previous studies on the stress concentrations of L. *vannamei* [[21], [22], [23]], to provide a reference for the control of the concentration of each chemical in the aquatic environment during subsequent shrimp aquaculture. In aquatic environments, the toxicity of ammonia nitrogen directly affects the survival of aquatic animals, resulting in reduced survival of shrimp [24].The results of this study showed that the three different concentrations of ammonia (A_1_ group: 10 mg/L ammonia-N, A_2_ group: 20 mg/L ammonia-N, A_3_ group: 30 mg/L ammonia-N) reduced the survival rate of shrimp compared to the control group, but there was no significant difference between the three concentrations of ammonia nitrogen on the survival rate of shrimp, probably because the gradient of ammonia nitrogen between the three concentrations was too small to have a significant effect on the mortality rate of shrimp.

Nitrite, as a common toxic substance, is widely present in aquatic systems, not only as a toxic intermediate produced during ammonia nitrification, but also as a product of bacterial denitrification of nitrate during the nitrogen cycle [25]. When nitrite exposure is prolonged, histologic effects may occur [18], and lead to high mortality. In the present study, the three concentrations of nitrite showed a gradual decrease in survival at the end of stress as the concentration increased, and the survival curves became progressively more different from those of the control group as the concentration of stress increased. The three nitrite concentrations were a greater threat to L. *vannamei* compared to ammonia and sulfide. This may be due to the high concentration of nitrite used in the experiment, or the stronger mechanism of toxicity of nitrite to L. *vannamei*, resulting in a lower tolerance of nitrite to the shrimp. The exact reason will be further explored in our follow-up study.

Both malondialdehyde (MDA) concentration and nitric oxide (NO) concentration were elevated in the hepatopancreas of L. *vannamei* under sulfide stress, while caspase-3 gene appeared to be up-regulated, indicating that the antioxidant system was disrupted and apoptosis was initiated [26]. In this study, the three concentrations of sulfide showed a gradual decrease in survival at the end of stress with increasing concentration. The survival curves of the S_3_ group were significantly different from both the control and S_1_ groups, indicating that the survival of the shrimp was more affected as the sulfide concentration increased. With increasing sulfide concentration, a significant reduction was observed in the hyaline cell count, total hemocyte count, phenoloxidase activity, phagocytic activity, and bacterial clearance efficiency of L. *vannamei* [27]. These physiological and immune responses likely contributed to the gradual decline in survival rate as sulfide concentration increased.

### Effects of various concentrations of different chemicals on the morphology of hepatopancreatic tissue of L. *vannamei*

Hepatopancreas is the largest immune organ of shrimp. Its main function is digestion, absorption and storage of nutrients, and plays a key role in maintaining the metabolic balance of shrimp and eliminating toxic pollutants [28]. In the present study, significant damage was observed in the hepatopancreas under the stress of the three environmental factors, which may be attributed to the greater burden on the hepatopancreas in balancing and clearing the pollutants, resulting in damage to its tissue structure.

In the present study, all the three different chemicals caused damage to hepatopancreatic tissues of L. *vannamei*. As the concentration of ammonia nitrogen increased, the cellular deformation gradually increased and the number of cellular vacuolization gradually increased. In previous studies, 10 mg/L ammoniacal nitrogen caused full-size vesicles in the hepatopancreas with blurred boundary lines between cells, and 20 mg/L ammoniacal nitrogen caused severe lysis of hepatopancreatic cells in L. *vannamei*, with connective tissue congestion and hemolymphocytic infiltration observed [22]. The present study is in general agreement with the results of previous studies, indicating that hepatopancreatic injury progressively worsened with increasing ammonia nitrogen concentration. Hepatopancreas of L. *vannamei* was damaged under nitrite stress, with cell shrinkage, severe lysis of some epithelial cells of the hepatic tubules, and apparent vacuolization [10]. In this study, all the three different concentrations of nitrite caused extensive necrosis of the hepatopancreas and severe lysis of hepatopancreatic cells, indicating that all the three concentrations of nitrite stress for 132 h caused severe damage to the hepatopancreas. Under sulfide stress, the hepatopancreas of *Charybdis japonica* showed irregular epithelial morphology, increased vesicles, and swelling and deformation of the rough endoplasmic reticulum [29]. However, there are fewer reports on the effects of different concentrations of sulfide on the hepatopancreatic tissue structure of L. *vannamei*. In the present study, we found that three different concentrations of sulfide gradually dispersed the cellular arrangement and the number of cell shrinkage and deformation increased with the increase of the concentration, which indicated that the higher the concentration of sulfide, the more serious the damage to the hepatopancreas. The present study provides a reference for the subsequent effects of sulfide on various aspects of the hepatopancreas of L. *vannamei*.

In the present study, the hepatopancreas was significantly more severely damaged under nitrite stress than under ammonia and sulfide stress. There was no significant difference in hepatopancreatic injury under stress between group A_1_ vs. group S_1_, group A_2_ vs. group S_2_, and group A_3_ vs. group S_3_. This may be due to the fact that all the three concentrations of nitrite caused severe damage to the hepatopancreas of L. *vannamei* under stress for 132 h, and it is also possible that nitrite is more susceptible to hepatopancreatic damage in L. *vannamei* compared to ammonia and sulfide. Hepatopancreatic tissues are more severely damaged under nitrite stress, which may have a greater impact on immune function and metabolic system of shrimp, and the effects of different concentrations of chemicals on the hepatopancreatic system will subsequently be fully assessed in conjunction with transcriptomic and metabolomic analyses.

### Effects of various concentrations of different chemicals on the morphology of midgut tissue of L. *vannamei*

The intestinal tract of aquatic animals is an important organ for nutrient absorption and immunity, and the intestinal barrier affects the health of shrimp [30,31]. The intestinal barrier is the first line of defense against pathogen infections and environmental stresses in shrimp, which is related to its structural integrity, microbial composition, and mucosal immune compounds [21]. The integrity and inflammatory state of the animal gut has been used to assess the intestinal health of animals [32].

Typically, the normal gut has tightly arranged epithelial cells, well-defined cell gaps, columnar epithelial tissue lining and structurally intact villi. Disruption of the intestinal mucosal structure increases the risk of pathogen invasion of the host, thereby disrupting host immunity. In a previous study, the intestines of L. *vannamei* exposed to ammonia nitrogen and nitrite, respectively, for 72 h showed histological damage features such as epithelial detachment from the basement membrane, loosening of junctions, necrosis of a large portion of the epithelium, formation and rupture of vacuoles, and scattering of nuclei in the lumen of the intestine, as compared to the control group [33]. In the present study, all the three different concentrations of ammonia nitrogen caused damage to the midgut of L. *vannamei*, with fragmentation and breakage of the epithelial cells, which is consistent with the results of the previous study, indicating that ammonia nitrogen causes damage to the midgut of L. *vannamei*. In this study, the separation of epithelial cells from the basement membrane gradually increased with increasing ammonia nitrogen concentration, suggesting that changes in ammonia nitrogen concentration affect the degree of separation of epithelial cells. In the present study, all the three nitrites damaged the midgut, with dead epithelial cells disrupted and connections broken, which is consistent with the results of previous studies indicating that nitrite causes damage to the midgut. Meanwhile, the degree of separation of epithelial cells from the basement membrane was significantly more severe in group Y_3_ than in group Y_1_ and group Y_2_, suggesting that high concentrations of nitrite increase the degree of separation. Part of the intestine of shrimp exposed to 425.5 μg/L sulfide had gaps between the intestine and epithelial cells, and some of the intestinal epithelial cells were separated from the basement membrane. The intestinal epithelial cells of shrimp exposed to the high sulfide concentration of 851 μg/L were completely separated from the basement membrane. The total number of intestinal epithelial cells and the height of microvilli tended to decrease with increasing sulfide concentration [21]. In the present study, all the three concentrations of sulfide caused damage to the midgut, with epithelial cell shrinkage and slight separation from the basement membrane in group S_1_, and increased epithelial cell fragmentation and separation from the basement membrane in group S_2_ and group S_3_. This is in line with previous findings suggesting a progressive increase in damage to the midgut of L. *vannamei* with increasing sulfide concentrations. In this study, it is known that all the three chemicals caused damage to the intestinal tissues of the shrimp, and the destruction of the intestinal tract increases the susceptibility of the pathogens [21], so the three chemicals will increase the susceptibility of the shrimp to which diseases under stress will need to be further investigated.

In the present study, all the three chemicals caused damage to the midgut of L. *vannamei*, with necrotic fragmentation of epithelial cells and some separation of epithelial cells from the basement membrane occurring in all of them. Between the three chemicals, the degree of detachment of epithelial cells from the basement membrane appeared to be more severe in group Y_1_ than in groups A_1_ and S_1_, more severe in group Y_2_ than in groups A_2_ and S_2_, and more severe in group Y_3_ than in groups A_3_ and S_3_, which indicates that these three concentrations of nitrite (20 mg/L, 40 mg/L, 60 mg/L) cause more damage to the midgut than ammonia (10 mg/L, 20 mg/L, 30 mg/L) and sulfide (2 mg/L, 3 mg/L, 4 mg/L). The midgut is a key organ for digestion and nutrient absorption in shrimp, and disruption of its structure may affect secretion of digestive enzymes. The ability to secrete digestive enzymes may be correlated with tissue structural damage, and digestive enzymes may be weakened with increasing concentrations of the three environmental factors, with nitrite stress potentially weakening the ability to secrete digestive enzymes more than ammonia and sulfide stress. Future studies may further validate these speculations by examining digestive enzyme activity, nutrient uptake efficiency, and the expression of metabolism-related genes.

### Effects of various concentrations of different chemicals on the morphology of muscle tissue of L. *vannamei*

High meat quality is an important motivation for consumers to buy, so muscle quality of L. *vannamei* is critical in shrimp farming. Studies have shown that collagen in muscle is closely related to meat quality [34]. Collagen is a fibrous protein made up of three peptide chains and is an important component of connective tissue. Myofibers are tightly connected to the perimysium and endomysium, which are made up of collagen, so that the muscle has certain textural properties [35].

Shrimp muscle undergoes severe lipid peroxidation and myofibrillar protein (MP) oxidative degradation when exposed to ammoniacal nitrogen, which may unfold the protein structure and weaken muscle hardness [36], impairing the integrity of myofibrils in the muscle tissues, thereby reducing hardness and water retention capacity. In the present study, all the three concentrations of ammonia nitrogen caused muscle fibers to break, which is consistent with previous studies, suggesting that ammonia nitrogen can cause muscle damage and impair the integrity of myofibers in muscle tissue. The gap between adjacent muscle bundles was larger in A_3_ group than in A_1_ and A_2_ groups, indicating that the high concentration of ammonia nitrogen was more serious for muscle damage. However, there are relatively limited studies on the muscle of L. *vannamei* under nitrite and sulfide stress. In the present study, all the three concentrations of nitrite caused the muscle to break into pieces, with uneven distribution of nuclei and nuclear agglutination, indicating that all the three concentrations of nitrite caused severe damage to the muscle. The muscles under three different concentrations of sulfide stress underwent muscle fiber fragmentation and rupture, and the degree of muscle fragmentation in the S_3_ group was more serious than that in the S_1_ and S_2_ groups, indicating that the higher the concentration of sulfide, the greater the damage to the muscle.

In this study, nitrite was overall more damaging to muscle than ammonia and sulfide. Muscle fragmentation was more severe in group Y_1_ than in groups A_1_ and S_1_, and separation between adjacent muscle bundles was more pronounced in group Y_2_ than in groups A_2_ and S_2_. The muscles in the A_3_, Y_3_, S_3_ groups were severely damaged, with muscle fibers severely torn and tissue torn into blocks. Muscle tissue is a critical site for the locomotor ability of shrimp, energy storage and protein metabolism, and damage to its structure may have a significant impact on its function. Tissue damage was more severe under the stress of high concentrations of ammonia, nitrite and sulfide, and the fracture and necrosis of muscle fibers may weaken the shrimp's movement, and the severe tissue damage may further affect the muscle protein synthesis and repair ability. These hypotheses can be further verified in future studies by examining the expression of genes related to exercise performance, energy metabolism-related enzymes and muscle protein synthesis.

### Effects of various concentrations of different chemicals on the morphology of gill tissue of L. *vannamei*

The gills of crustaceans play a key role in both respiration and the regulation of homeostasis in the body [37]. The gill is an important respiratory organ of shrimp and is the main target organ for the toxic effects of nitrite, as well as functions such as osmoregulation and nitrogen excretion, and is also involved in the immune response to clear pathogens [38].

Nitrogen compounds such as ammonia and nitrite cause damage to gill structures, leading to tissue hypoxia and affecting acid-base balance, osmoregulation and ammonia excretion [39]. The present study is in agreement with previous studies that both ammonia and nitrite caused damage to gill tissues. In the present study, the gill filaments gradually became distorted from neatly arranged and the contraction and deformation gradually increased with the increase of ammonia nitrogen concentration, indicating that the damage to the gills gradually increased with the increase of ammonia nitrogen concentration. Three concentrations of nitrite resulted in progressively greater gill filament contraction and progressive thinning and rupture of the cuticle as the concentration increased, indicating progressively greater damage to the gill filaments at higher concentrations of nitrite. Short-term acute sulfide damages the morphological structure of the gills of L. *vannamei*, affecting its osmoregulation, respiratory metabolism, and immune status [40]. In the present study, the gill filaments gradually became disorganized from slight twisted deformations and the gill constriction gradually worsened with the increase in sulfide concentration. In group S_3_, the gill vessels were severely deformed, and the entering gill vessels and exiting gill vessels were blurred, indicating that the higher the concentration of sulfide, the more serious the damage to the gill filaments.

In the present study, nitrite was overall more damaging to gill filaments than ammonia and sulfide. Groups A_1_ and S_1_ had neatly arranged gill filaments with little damage and no significant differences, while group Y_1_ showed disorganized gill filaments with obvious contractions. In groups A_2_ and S_2_, the gill filament arrangement appeared slightly disturbed and some gill filaments were contracted and deformed. In group Y_2_, gill filament contraction was obvious, the number of hemocytes was increased, and the cuticle appeared to be broken. The gill filaments of groups A_3_, Y_3_ and S_3_ were severely damaged, with severe contraction and deformation and blurred gill vessels. Under high concentrations of ammonia, nitrite, and sulfide stress, the damage to the gill filaments is more severe, which may lead to more significant impairment of their ion regulation function. The severe tissue damage caused by the high concentrations of the three stressors may further impair the excretory function of gills and lead to the accumulation of toxic metabolites. Future research could further validate these hypotheses by detecting the activity of ion regulation-related enzymes and the expression of genes associated with excretory function in the gills.

## Conclusions

The present study investigated the impact of ammonia, nitrite, and sulfide stress on L. *vannamei*, focusing on survival and tissue morphology. The results of the study showed that all the three chemicals reduced the survival rate of shrimp, and the damage to tissues by all the three chemicals gradually increased with the increase of stress concentration, with nitrite showing overall more severe tissue damage than ammonia and sulfide. These results underscore the importance of monitoring and managing water quality in aquaculture systems to mitigate the adverse effects of these common environmental stressors. Future studies will further explore the molecular and physiological mechanisms underlying these responses to develop targeted strategies for improving shrimp resilience.

## CRediT authorship contribution statement

**Lulu Han:** Writing – original draft, Methodology, Investigation. **Peiyu Yan:** Project administration, Investigation. **Mengqiang Wang:** Writing – review & editing, Supervision, Project administration, Funding acquisition, Conceptualization.

## Declaration of competing interest

The authors declared that they have no conflicts of interest to this work. And all the authors have approved the manuscript and agree with this submission.

## Data Availability

Data will be made available on request.

## References

[1] Asche F., Anderson J.L., Botta R., Kumar G., Abrahamsen E.B., Nguyen L.T., Valderrama D. (2021). The economics of shrimp disease. J. Invertebr. Pathol..

[2] Millard R.S., Ellis R.P., Bateman K.S., Bickley L.K., Tyler C.R., van Aerle R., Santos E.M. (2021). How do abiotic environmental conditions influence shrimp susceptibility to disease? A critical analysis focussed on White Spot disease. J. Invertebr. Pathol..

[3] Chan M.W.H., Ali A., Ullah A., Mirani Z.A., Balthazar-Silva D. (2021). A size-dependent bioaccumulation of metal pollutants, antibacterial and antifungal activities of telescopium telescopium, Nerita albicilla and Lunella coronata. Environ. Toxicol. Pharmacol..

[4] Chan M.W.H., Hasan K.A., Balthazar-Silva D., Mirani Z.A., Asghar M. (2021). Evaluation of heavy metal pollutants in salt and seawater under the influence of the Lyari River and potential health risk assessment. Mar. Pollut. Bull..

[5] Aslam S., Chan M.W.H., Siddiqui G., Boczkaj G., Kazmi S.J.H., Kazmi M.R. (2020). A comprehensive assessment of environmental pollution by means of heavy metal analysis for oysters' reefs at Hab River Delta. Mar. Pollut. Bull..

[6] Hussain Chan M.W., Hasan K.A., Balthazar-Silva D., Asghar M., Mirani Z.A. (2021). Surviving under pollution stress: antibacterial and antifungal activities of the Oyster species (Magallana bilineata and Magallana cuttackensis). Fish Shellfish Immunol.

[7] Xiao J., Liu Q.Y., Du J.H., Zhu W.L., Li Q.Y., Chen X.L., Chen X.H., Liu H., Zhou X.Y., Zhao Y.Z., Wang H.L. (2020). Integrated analysis of physiological, transcriptomic and metabolomic responses and tolerance mechanism of nitrite exposure in Litopenaeus vannamei. Sci. Total Environ..

[8] Qin C.J., Shao T., Wang Y.M., Gong Q., Yang Q., Bu P. (2017). Effect of ammonia-N on histology and expression of immunoglobulin M and component C3 in the spleen and head kidney of Pelteobagrus vachellii. Aquac. Rep..

[9] Rostami F., Davoodi R., Nafisi Bahabadi M., Salehi F., Nooryazdan H. (2019). Effects of ammonia on growth and molting of Litopenaeus vannamei postlarvae reared under two salinity levels. J. Appl. Aquacult..

[10] Yang S., Luo J., Huang Y., Yuan Y., Cai S. (2022). Effect of sub-lethal ammonia and nitrite stress on autophagy and apoptosis in hepatopancreas of Pacific whiteleg shrimp Litopenaeusvannamei. Fish Shellfish Immunol.

[11] Cui Y., Ren X., Li J., Zhai Q., Feng Y., Xu Y., Ma L. (2017). Effects of ammonia-N stress on metabolic and immune function via the neuroendocrine system in Litopenaeus vannamei. Fish Shellfish Immunol.

[12] Xue S., Chen S., Ge Y., Guan T., Han Y. (2022). Regulation of glutathione on growth performance, biochemical parameters, non-specific immunity, and related genes of common carp (Cyprinus carpio) exposed to ammonia. Aquaculture.

[13] Ou H., Liang J., Liu J. (2022). Effects of acute ammonia exposure on oxidative stress, endoplasmic reticulum stress and apoptosis in the kuruma shrimp (Marsupenaeus japonicus). Aquac. Rep..

[14] Huang W., Yin H., Yang Y., Jin L., Lu G., Dang Z. (2021). Influence of the co-exposure of microplastics and tetrabromobisphenol A on human gut: simulation in vitro with human cell caco-2 and gut microbiota. Sci. Total Environ..

[15] Huang M., Xie J., Yu Q., Xu C., Zhou L., Qin J.G., Chen L., Li E. (2020). Toxic effect of chronic nitrite exposure on growth and health in Pacific white shrimp Litopenaeus vannamei. Aquaculture.

[16] Xiao J., Luo S.S., Du J.H., Liu Q.Y., Huang Y., Wang W.F., Chen X.L., Chen X.H., Liu H., Zhou X.Y., Zhao Y.Z., Wang H.L. (2022). Transcriptomic analysis of gills in nitrite-tolerant and -sensitive families of Litopenaeus vannamei. Comp. Biochem. Physiol. C: Pharmacol. Toxicol..

[17] Yang S., Luo J., Huang Y., Yuan Y., Cai S. (2022). Effect of sub-lethal ammonia and nitrite stress on autophagy and apoptosis in hepatopancreas of Pacific whiteleg shrimp Litopenaeus vannamei. Fish Shellfish Immunol.

[18] Lin L., Zhang Y., Zhuo H., Li J., Fu S., Zhou X., Wu G., Guo C., Liu J. (2024). Integrated histological, physiological, and transcriptome analysis reveals the post-exposure recovery mechanism of nitrite in Litopenaeus vannamei. Ecotoxicol. Environ. Saf..

[19] Vismann B. (2012). Sulfide tolerance: physiological mechanisms and ecological implications. Ophelia.

[20] Duan Y., Dong H., Wang Y., Li H., Liu Q., Zhang Y., Zhang J. (2017). Intestine oxidative stress and immune response to sulfide stress in Pacific white shrimp Litopenaeus vannamei. Fish Shellfish Immunol.

[21] Suo Y., Li E., Li T., Jia Y., Qin J.G., Gu Z., Chen L. (2017). Response of gut health and microbiota to sulfide exposure in Pacific white shrimp Litopenaeus vannamei. Fish Shellfish Immunol.

[22] Si L., Pan L., Wang H., Zhang X. (2019). Ammonia-N exposure alters neurohormone levels in the hemolymph and mRNA abundance of neurohormone receptors and associated downstream factors in the gills of Litopenaeus vannamei. J. Exp. Biol..

[23] Li Z.S., Ma S., Shan H.W., Wang T., Xiao W. (2019). Responses of hemocyanin and energy metabolism to acute nitrite stress in juveniles of the shrimp Litopenaeus vannamei. Ecotoxicol. Environ. Saf..

[24] Zhuo H., Zhang Y., Fu S., Lin L., Li J., Zhou X., Wu G., Guo C., Liu J. (2024). miR-8-3p regulates the antioxidant response and apoptosis in white shrimp, Litopenaeus vannamei under ammonia-N stress. Int. J. Biol. Macromol..

[25] Tomasso J.R. (2012). Environmental nitrite and aquaculture: a perspective. Aquacult. Int..

[26] Jiang L., Feng J., Ying R., Yin F., Pei S., Lu J., Cao Y., Guo J., Li Z. (2019). Individual and combined effects of ammonia-N and sulfide on the immune function and intestinal microbiota of Pacific white shrimp Litopenaeus vannamei. Fish Shellfish Immunol.

[27] Hsu S.W., Chen J.C. (2007). The immune response of white shrimp Penaeus vannamei and its susceptibility to Vibrio alginolyticus under sulfide stress. Aquaculture.

[28] Frías-Espericueta M.G., Bautista-Covarrubias J.C., Osuna-Martínez C.C., Delgado-Alvarez C., Bojórquez C., Aguilar-Juárez M., Roos-Muñoz S., Osuna-López I., Páez-Osuna F. (2022). Metals and oxidative stress in aquatic decapod crustaceans: A review with special reference to shrimp and crabs. Aquat. Toxicol..

[29] Xu X.H., Zhang Y.Q., Yan B.L., Xu J.T., Tang Y., Du D.D. (2014). Immunological and histological responses to sulfide in the crab Charybdis japonica. Aquat. Toxicol..

[30] Gao C., Fu Q., Su B., Zhou S., Liu F., Song L., Zhang M., Ren Y., Dong X., Tan F., Li C. (2016). Transcriptomic profiling revealed the signatures of intestinal barrier alteration and pathogen entry in turbot (Scophthalmus maximus) following Vibrio anguillarum challenge. Dev. Comp. Immunol..

[31] Rungrassamee W., Klanchui A., Maibunkaew S., Karoonuthaisiri N. (2016). Bacterial dynamics in intestines of the black tiger shrimp and the Pacific white shrimp during Vibrio harveyi exposure. J. Invertebr. Pathol..

[32] Bischoff S.T. (2011). Lexical affixes, incorporation, and conflation: the case of Coeur d'Alene. Stud. Linguistica.

[33] Duan Y., Liu Q., Wang Y., Zhang J., Xiong D. (2018). Impairment of the intestine barrier function in Litopenaeus vannamei exposed to ammonia and nitrite stress. Fish Shellfish Immunol.

[34] Hagen Ø., Johnsen C.A. (2016). Flesh quality and biochemistry of light-manipulated Atlantic cod (Gadus morhua) and the significance of collagen cross-links on fillet firmness and gaping. Food Chem.

[35] Ye T., Xiang Q., Yang Y., Huang Y. (2023). Research, development and application of collagen: a review. Chin. J. Biotechnol..

[36] Hematyar N., Rustad T., Sampels S., Dalsgaard T.Kastrup (2019). Relationship between lipid and protein oxidation in fish. Aquacult. Res..

[37] McNamara J.C., Faria S.C. (2012). Evolution of osmoregulatory patterns and gill ion transport mechanisms in the decapod Crustacea: a review. J. Comp. Physiol. B.

[38] Xing Y., Zhu X., Duan Y., Huang J., Nan Y., Zhang J. (2023). Toxic effects of nitrite and microplastics stress on histology, oxidative stress, and metabolic function in the gills of Pacific white shrimp, Litopenaeus vannamei. Mar. Pollut. Bull..

[39] Dutra F.M., Rönnau M., Sponchiado D., Forneck S.C., Freire C.A., Ballester E.L.C. (2017). Histological alterations in gills of macrobrachium amazonicum juveniles exposed to ammonia and nitrite. Aquat. Toxicol..

[40] Duan Y., Wang Y., Dong H., Li H., Liu Q., Zhang J., Xiong D. (2018). Physiological and immune response in the gills of Litopenaeus vannamei exposed to acute sulfide stress. Fish Shellfish Immunol.

